# Isolated systolic hypertension: primary care practice patterns in a Nigerian high-risk subpopulation

**DOI:** 10.1590/S1516-31802006000200011

**Published:** 2006-03-02

**Authors:** Agbani Ejaife Ono, Erhun Wilson Oyekigho, Ojo Araoye Adeleke

**Keywords:** Hypertension, Primary health care, Blood pressure, Ambulatory blood pressure monitoring, Systole, Hipertensão, Cuidados primários de saúde, Pressão arterial, Monitorização ambulatorial da pressão arterial, Sístole

## Abstract

**CONTEXT AND OBJECTIVE:**

Hypertension management and risk prediction based on diastolic blood pressure may be of little value for older people and people with isolated systolic hypertension (ISH). This study investigated primary care practice patterns in ISH management in a Nigerian high-risk subpopulation.

**DESIGN AND SETTING:**

Three-year retrospective cohort review of outpatient medical records at a state primary health care facility in south-western Nigeria.

**METHODS:**

ISH was defined according to international guidelines. Treatments were graded as relatively non-aggressive, mildly aggressive and moderately aggressive. Data were collected using a data abstraction form and statistically analyzed.

**RESULTS:**

The drug/regimen choice controlled systolic blood pressure (SBP) in only 46.90% of the population after the first visit to the clinic. SBP control among treated patients was significantly inadequate. Group mean SBP was consistently > 150 mmHg in 28.13% of the patients for ≥ six weeks after enrollment and for at least two additional visits. Data analysis revealed an increasing tendency to place patients on monotherapy or "no drug treatment" with successive visits to the clinic, even in cases of uncontrolled systolic blood pressure, as well as declining prescription of moderately aggressive combination therapy.

**CONCLUSION:**

Aggressive ISH management needs to be further emphasized at primary care levels, which for many low-income patients may be the first and last orthodox port of call.

## RESUMO

**CONTEXTO E OBJETIVO:**

O controle da hipertensão e a previsão do grau de risco baseados na pressão diastólica podem ser considerados relativamente de pouco valor para pessoas idosas ou com hipertensão sistólica isolada (HSI). O presente estudo investigou padrões de controle da HSI em nível de cuidados primários em uma subpopulação nigeriana de alto risco.

**TIPO DE ESTUDO E LOCAL:**

Análise retrospectiva de três anos dos prontuários médicos de centro de saúde estadual de cuidados primários no sudoeste da Nigéria.

**MÉTODOS:**

A definição de hipertensão sistólica isolada (ISH) está de acordo com diretrizes internacionais. Tratamentos foram classificados como relativamente não-agressivos, ligeiramente agressivos e moderadamente agressivos. Os dados foram coletados usando um formulário de abstração de dados e analisados usando versão SPSS 11.0.

**RESULTADOS:**

A droga/regime de tratamento de escolha controlou a hipertensão arterial sistólica (HAS) em apenas 46.90% da população após a primeira visita. O controle da pressão arterial em pacientes tratados foi significativamente inadequado. A média da pressão arterial sistólica do grupo se manteve consistentemente acima de 150 mm Hg em 28.13% dos pacientes durante pelo menos seis semanas após haverem dado entrada na clínica e terem tido, pelo menos, duas visitas adicionais. A análise dos dados revelou tendência crescente de submeter os pacientes a monoterapia ou a "nenhuma terapia medicamentosa" com sucessivas visitas à clínica, mesmo em casos de hipertensão arterial sistólica descontrolada, bem como queda na prescrição de terapia combinada moderadamente agressiva.

**CONCLUSÃO:**

O controle da HSI agressiva necessita ser mais enfatizado no nível primário de cuidados de saúde, que, para muitos pacientes de baixa renda, podem significar o primeiro e último.

## INTRODUCTION

Hypertension management and risk prediction based on diastolic blood pressure may be reasonably valuable for younger people and people with essential hypertension. The use of diastolic blood pressure as a treatment yardstick has been supported by the discovery that essential hypertension is characterized by increased peripheral vascular resistance and raised mean arterial pressure, which more closely correlates with diastolic blood pressure (DBP) than with systolic blood pressure (SBP). However, data from cohort and intervention studies, as well as international guidelines, indicate that this practice is inappropriate for middle age and elderly hypertensive patients, particularly those with isolated systolic hypertension (ISH).^1,2^ Clinical trials have demonstrated that control of isolated systolic hypertension reduces total mortality, cardiovascular mortality, stroke and heart failure events.^2^ Both observational studies and clinical trial data suggest that poor SBP control is largely responsible for the unacceptably low rates of overall blood pressure control.^2^

Significant reduction in systemic arterial compliance is common with advancing age.^3^ This decrease in compliance results in higher systolic pressures, as the large vessels become less able to reduce the pressure generated by the left ventricle by means of distension. On the other hand, while increases in peripheral resistance will cause elevations in diastolic blood pressures, the loss of large-vessel elasticity does the opposite. Thus, with increasing age, these counteracting forces may keep the diastolic pressure normal while, in the background, there is increasing systolic pressure.^4-6^

Primary health care in Nigeria is heavily financed in many states and constitutes the first and perhaps the only port of call for orthodox care for many patients who suffer from chronic, often asymptomatic diseases like hypertension. This situation results from the peculiar burden of socioeconomic challenges. It becomes imperative, therefore, that disease management practice patterns at this level meet with present recommendations, especially among high-risk subpopulations.

The present study consists of a three-year retrospective review of the medical records of a state primary health care facility, among patients treated for ISH. The definition of ISH was in accordance with the sixth report from the American Joint National Committee on Hypertension (JNC-VI)^7^ and the World Health Organization (WHO) and International Society of Hypertension guidelines.^8^ These define ISH as SBP ≥ 140 mm Hg and DBP < 90 mmHg, and were the definitions available to the practitioners at the time of patient enrollments. The present study infers that, despite the recommendations in JNC-VI^7^ and subsequently in JNC-VII^2^ and other major studies,^9,10^ and their support for the importance of SBP, practitioners have tended to overlook and undertreat ISH at the primary care level.

## METHODS

### Design, setting and patients

This was a three-year retrospective cohort review of the outpatient medical records from a state primary health care facility in southwestern Nigeria. The study population were outpatients who were continuously registered at the health center between June 1999 and June 2002. These patients were aged 40 years or over, with history of hypertension lasting for nine months or more, and a minimum of six months of post-enrollment monitoring. Each subject's medical record was reviewed. The physicians at the health facility measured the patients' blood pressure by applying a cuff to the right arm and using a standard mercury sphygmomanometer.

The following data were collected using a data abstraction form:

- Demographics (age, gender and occupation);
- Comorbidities (history of myocardial infarction, diabetes, renal insufficiency, congestive heart failure, hyperlipidemia and stroke);
- Range of antihypertensive regimen within study period;
- Documented recommendations for lifestyle modification (salt-restricted diet, stress reduction, exercise programs, weight reduction and alcohol intake restriction).

ISH was defined in accordance with JNC-VI^7^ and WHO/International Society of Hypertension (1999):^8^ SBP ≥ 140 mm Hg and DBP < 90 mmHg. Accordingly, the different grades of ISH were defined as follows: stage 1: SBP < 160 mmHg, with the subgroup of borderline SBP < 150 mmHg; stage 2: SBP < 180 mmHg; and stage 3: SBP ≥ 180 mmHg.

Treatments were graded as relatively non-aggressive: no documented evidence of the prescription of anti-hypertensive or non-pharmacological treatment; mildly aggressive: monotherapy with either a centrally acting or a diuretic agent (not more than twice daily); and moderately aggressive: combination therapy with centrally acting agent and combination diuretics (not more than twice daily).

### Data analysis

Statistical analysis was done using the Statistical Package for the Social Sciences (SPSS) version 11.0 software (SPSS Inc., Chicago, United States). The test for statistical significance was by means of the chi-squared test for categorical data and one-way analysis of variance (ANOVA) for quantitative data. Cross-tabulation statistics and bivariate correlation were used to measure associations and investigate linear relationships between variables, respectively. Predictor and criterion modeling was done by means of bivariate regression analysis.

## RESULTS

The study population (n = 64) was composed of 31.3% males and 68.8% females, with a mean age of 57.41 years (standard deviation, SD = ± 10.95; standard error of the estimate, SEE = 1.37; skewness = 0.12); median age = 54.50 years; modal age = 60.00 years; minimum age = 40 years; and maximum age = 78. The patients resided at distances from the health facility ranging from 0.7 to around 50 km. The patients made their second, third and fourth visits to the clinic, 22.11 ± 14.55 (mode = 7), 42.00 ± 11.88 (mode = 51) and 52.33 ± 14.89 (mode = 42) days after enrollment. 21.9% of the patients were coprescribed either anti-malaria or anti-arthritis drugs along with the antihypertensive(s), at the time of their initial visit.

**Figure 1:**
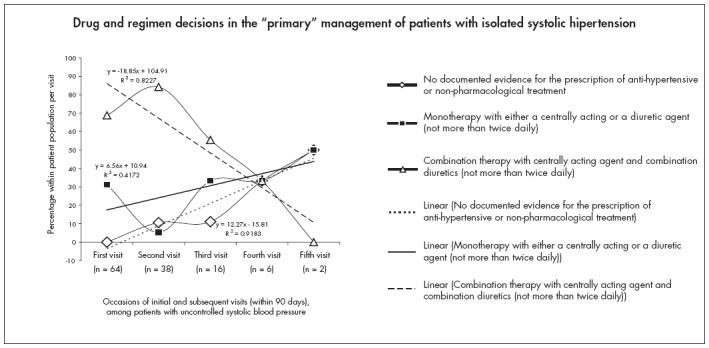
Drug and regimen decisions in the "primary" management of patients with isolated systolic hypertension.

The subjects presented with stage 1 ISH (34.4%), stage 1 borderline ISH (28.1%), stage 2 ISH (25.0%) and stage 3 ISH (12.5%). The caregiver's drug/regimen choice controlled SBP in only 46.90% of the population after the first visit. The group mean SBP was consistently greater than 150 mmHg in 28.13% of the patients for a minimum of six weeks after enrollment and for at least two additional visits to the clinic (Table 1). An attempt to simulate the trends in the prescription of monotherapy with either a centrally acting or a diuretic agent, or combination therapy with both, or the ignoring of elevated SBP, is shown in Figure 1. Data analysis by correlation and regression revealed an increasing tendency to place patients on monotherapy or "no drug treatment" with successive repeat visits to the clinic, even in cases of uncontrolled SBP, as well as declining prescription of moderately aggressive combination therapy as patients revisited the clinic (Figure 1 and Table 1). ISH patients who received "no drug treatment" on occasions after enrollment were either in borderline stage 1 ISH (33.3%) or in stage 1 ISH (66.7%).

**Table 1:** Clinical outcome in the management of isolated systolic hypertension in Nigerian blacks at the primary care level

	Systolic blood pressure in mmHg (first visit)	Diastolic blood pressure in mmHg (first visit)	Systolic blood pressure in mmHg (second visit)	Diastolic blood pressure in mmHg (second visit)	Systolic blood pressure in mmHg (third visit)	Diastolic blood pressure in mmHg (third visit)	Systolic blood pressure in mmHg (fourth visit)	Diastolic blood pressure in mmHg (fourth visit)
Mean	154.06	83.97	151.05	83.40	155.00	83.72	133.33	73.33
Median	150.00	85.00	160.00	85.00	160.00	80.00	130.00	70.00
Mode	150.00	80.00	160.00	80.00	160.00	80.00	120.00*	70.00
Standard deviation	12.50	3.90	20.77	4.13	10.85	4.30	13.66	5.16
Variance	156.25	15.21	431.30	17.07	117.65	18.44	186.67	26.67
Skewness	0.90	0.06	-0.57	-0.15	0.156	0.27	0.52	0.97
Standard error of skewness	0.30	0.30	0.38	0.38	0.536	0.54	0.85	0.85
Range	40.00	9.00	80.00	14.00	35.00	9.00	30.00	10.00
Minimum	140.00	80.00	100.00	75.00	140.00	80.00	120.00	70.00
Maximum	180.00	89.00	180.00	89.00	175.00	89.00	150.00	80.00

* *Multiple modes exist. The smallest value is shown*.

Comparison of the means and one-way ANOVA showed a significant difference in patients' SBP from the first visit to the clinic to the second visit (p = 0.000; F = 7.011, two-tailed). However, this difference became insignificant when the first visit was compared with the third (p = 0.062; F = 2.940), and for the second and third revisit (p = 0.824; two-tailed). We found a linear relationship that fluctuated in strength, between patients' SBP and the graded levels of treatment aggressiveness [first visit: p = 0.00, r^2^ = 0.695, r = 0.834, SEE = 0.846; second visit: p = 0.550, r^2^ = 0.010, r = 0.100, SEE = 20.95; third visit: p = 0.003, r^2^ = 0.441, r = 0.664, SEE = 8.36; fourth visit: p = 0.231, r^2^ = 0.332, r = 0.577, SEE = 12.481]. Bivariate regression analysis performed to model the interactions between these variables revealed that the number of visits to the clinic linearly predicted 66.6% of the variances in drug/regimen decisions during the study period (r = 0.82, SEE = 2.42, p < 0.01).

## DISCUSSION

Works by Cooper et al.,^11^ Bovet et al.,^12^ Cappuccio et al.^13^ and Erhun et al.^14^ have reported on the prevalence, awareness, treatment and control of hypertension in western Africa and comparable populations. Cooper et al.^11^ reported that the hypertension prevalence rate was 14.5%, while Erhun et al.^14^ reported a raw prevalence rate of 21% in a workplace study of hypertension prevalence amongst Nigerians. However, there appears to be a paucity of data on the prevalence of isolated systolic hypertension (ISH) among Nigerian blacks, using the JNC-VI^7^ and 1999 WHO/ International Society of Hypertension^8^ definitions. Nonetheless, the disease remains the most common type of hypertension and the most prevalent type of untreated hypertension among the elderly.^15^

The present study shows that no drug treatment was being implemented in 10.6%, 11.1%, 33.0% and 50% of the study patients returning with uncontrolled systolic blood pressure (SBP) at the second, third, fourth and fifth visits to the clinic, respectively (Figure 1 and Table 1). The drug therapies in use on these occasions were largely non-aggressive and non-individualized mono or combination therapy (Table 1). Blood pressure control among the treated patients was significantly inadequate (p > 0.05) until after the patients' second visit to the clinic. The mean and modal SBP remained ≥ 150 mmHg for periods estimated to be between one and six weeks after the patients' initial enrollment into the clinic (Table 1). We wonder how these patients survived the study period without any documented evidence of complications.

Isolated systolic hypertension increases cardiovascular or cerebrovascular morbidity and all-cause mortality twofold or more and triples cardiovascular mortality.^16^ Trials have established that systolic blood pressure is a stronger predictor of outcome than diastolic blood pressure, and that an excess risk of cardiovascular diseases exists in subjects with stage 1 (borderline) ISH.^16-20^ A significant number (p < 0.01; Figure 1) of such patients remained untreated during the study period. Untreated ISH patients show a high prevalence of left ventricular hypertrophy through concentric remodeling,^21^ and this has been shown to have a poor cardiovascular prognosis.^22^ On the other hand, the trends of drug/regimen decisions, practice patterns and consequent clinical outcomes observed in this study leave much to be done in the management of these patients. The international guidelines for the management of ISH^7,8^ that were available to the physicians during the study period recommend lifestyle modifications (physical exercise, sodium restriction and weight reduction in obese patients) as the first-line therapy for patients with ISH. The authors have found no documented evidence of this, although the current guide- lines^2^ indicate stiffer measures. JNC-VII^2^ classifies the modal and median ages of the study patients as indicative for the application of "sooner and tougher" measures, with lower target blood pressure, since at this age systolic blood pressure (> 140 mmHg) is much more important than high diastolic pressure as a risk factor for cardiovascular events^2^ and, beginning at 115 mmHg, the risk of cardiovascular disease doubles with each increment of 20 mmHg.

Overreliance on the importance of diastolic blood pressure (DBP) and the largely unsubstantiated concerns about the potential adverse consequences of treating SBP are perhaps the major reasons for the continuing reluctance to accept ISH as a discrete pathological entity, despite the established benefits from treating this disorder.^9,10^ Moreover, most physicians have been taught that diastolic blood pressure is more important than SBP and thus treat accordingly.^2^ Poor SBP control is at least in part related to physicians' attitudes.^2^ A survey of primarycare physicians indicated that three-fourths of them failed to initiate antihypertensive therapy in older individuals with SBP of 140-159 mmHg, and most primary-care physicians did not pursue control to levels of below 140 mmHg.^23,24^ Strict adherence to current guidelines will forestall the practice of "no drug treatment", in the way that was common among the patients of the present study who had borderline stage 1 ISH at their revisits to the clinic (Figure 1 and Table 1).

Evidence from clinical trials on antihypertensives published between January 1995 and December 2002 shows that low-dose diuretics are the most effective first-line treatment for preventing the occurrence of cardiovascular disease morbidity and mortality,^25^ and that most patients with hypertension will require two or more antihypertensive medications to achieve the target blood pressure. Drug/regimen and non-pharmacological management should therefore progressively become aggressive as the blood pressure of returning patients remains uncontrolled, as opposed to the increasing tendency to place patients on monotherapy or "no drug treatment" with successive repeat visits to the clinic, even in cases of uncontrolled SBP, as well as declining prescription of moderately aggressive combination therapy as patients revisited the clinic, observed in our study (Figure 1 and Table 1). This, in addition to the practice of prescribing centrally-acting agents (perhaps because this is cheaper), needs to be reviewed in the light of current guidelines and recommendations. In the recently published Antihypertensive and Lipid-Lowering Treatment to Prevent Heart Attack Trial (ALLHAT),^26^ more than 33,000 patients with hypertension were randomly assigned to receive amlodipine, lisinopril or chlorthalidone. The thiazide-type diuretic was shown to be most effective in controlling systolic blood pressure, as well as for preventing heart failure and stroke; it was also the least costly.

The present study has involved the analysis of secondary data, while certain factors such as the mechanism for enhancing or monitoring patients' compliance during treatment remained unclear. Notwithstanding this, caregivers at the primary care level need to identify therapeutic, cultural, educational, social and environmental factors that may impede the attainment of recommended treatment goals, so as to provide workable cost-effective interventions.

## CONCLUSIONS

Isolated systolic hypertension should be seen as an important clinical condition and aggressively managed at the primary care level, which, for most patients in the setting of the present study, may be the first and last orthodox port of call.
